# Three Water Molecules Mediate Ring Opening of d‑Glucose in Aqueous Solutions

**DOI:** 10.1021/acsphyschemau.6c00055

**Published:** 2026-06-09

**Authors:** Mawuli Deegbey, Valerie Vaissier Welborn

**Affiliations:** † Department of Chemistry, 1757 Virginia Tech, Blacksburg, Virginia 24061, United States; ‡ Macromolecules Innovation Institute, Virginia Tech, Blacksburg, Virginia 24061, United States

**Keywords:** Carbohydrates, Proton Transfer, Mutarotation, Free Energy, Water-bridge, Activation Barrier

## Abstract

The mechanism of d-glucose ring opening has long been debated, particularly with regard to whether proton transfer occurs through a concerted or stepwise pathway and how many water molecules participate in the process. Here, we employ ab initio molecular dynamics combined with metadynamics to investigate both possibilities. Our results identify a lower-energy concerted pathway in which proton transfer is mediated by a three-water hydrogen-bond network. Indeed, the transition state features a cyclic hydrogen-bonded water bridge linking OH_1_ and O_5_. This bridge promotes proton transfer from OH_1_ to O_5_, thereby initiating ring opening via an asynchronous yet concerted mechanism. This study provides the first direct dynamical evidence for the involvement of a three-water cyclic network in d-glucose ring opening, in support of earlier mechanistic proposals. The computed activation barrier of 15–18 kcal mol^–1^ agrees closely with experimental estimates.

## Graphical abstract

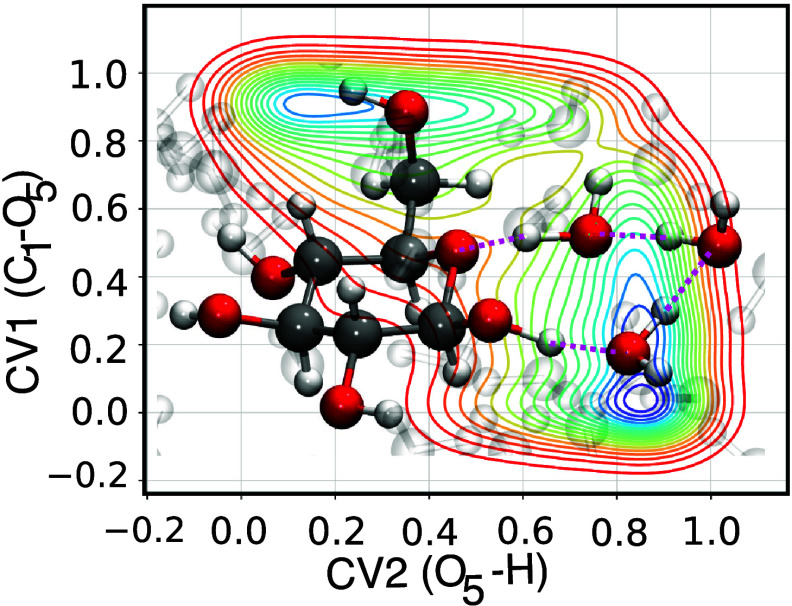

## Introduction

d-Glucose is a ubiquitous monosaccharide that plays a central role in energy metabolism across all forms of life and serves as a critical biomarker for human health.^1–4^ In water, d-glucose is found predominantly in its cyclic (hemiacetal) form, which undergoes anomeric equilibration between α- and β-d-glucose.^5,6^ Experiments show that this equilibrium favors the β-anomer (64%), with the α-anomer comprising the remaining 36%.^7–9^ Despite differing only by the orientation of a single hydroxyl group, the two anomers exhibit distinct physicochemical properties. For example, polymerization of α-d-glucose via (1 → 4) glycosidic linkages yields amylose, a helical polysaccharide that is a major constituent of starch and serves as an energy reserve in plants. By contrast, polymerization of β-d-glucose through the same (1 → 4) linkage produces cellulose, a linear, rigid polysaccharide that forms microfibrils and provides structural integrity to plant cell walls.^10–12^

Mutarotation, the spontaneous interconversion between α- and β-d-glucose, is a fundamental process in carbohydrate chemistry.^13–16^ Mutarotation proceeds through ring opening of the cyclic anomers to give the thermodynamically less stable acyclic aldehyde form of d-glucose. This acyclic form also serves as an intermediate in other transformations, including protein glycation (Figure 1). In this case, the carbonyl group reacts with a primary amino group in lysine or arginine residues to form a Schiff base.^17,18^ The Schiff base then undergoes an intramolecular rearrangement to produce a more stable ketoamine known as the Amadori product. Over time, and under appropriate conditions, Amadori products undergo further rearrangements and oxidative reactions to generate advanced glycation end-products (AGEs).^19,20^ The accumulation of AGEs has been linked to the development of a range of conditions, including diabetes,^21,22^ Alzheimer’s disease,^23,24^ aging,^25,26^ and various skin and bone disorders.^27–29^ Since ring opening is a necessary step in many processes, mutarotation has long been used as a model system for reactions that proceed through acyclic d-glucose intermediates.^30–42^ However, this intermediate is highly reactive, and is therefore present only in trace quantities at equilibrium - typically accounting for less than 1% of d-glucose in aqueous solution.^8,9,13,43^ Its low abundance makes direct experimental characterization challenging and complicates detailed mechanistic analysis.

**Figure 1:**
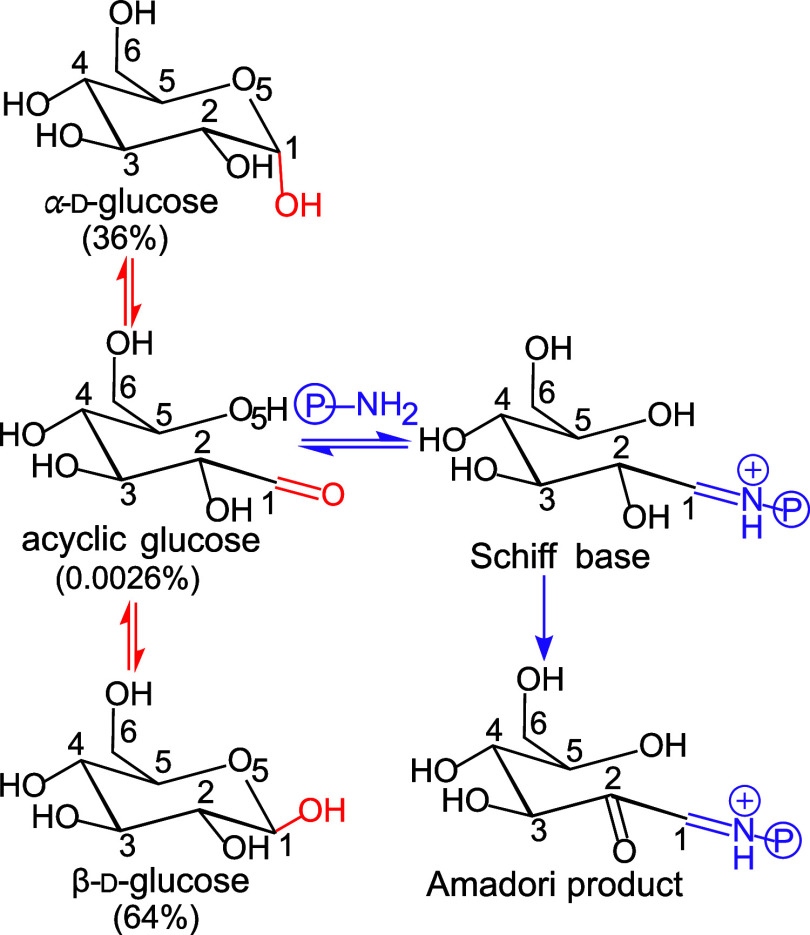
Role of acyclic d-glucose in mutarotation (red pathway) and early protein glycation (purple pathway). The axial and equatorial orientation of the OH groups are shown in red, and the protein with amino group (NH_2_) is shown in purple.

Kinetic investigations over the twentieth century established that mutarotation occurs under acidic, basic, and neutral conditions, but proceeds more rapidly in acidic or basic media than in pure water.^44–46^ Further, no significant rate difference was found between α- and β-d-glucose, indicating that the reaction environment exerts a stronger influence than the identity of the anomer.^13,14,45,47^ Activation barriers measured in mixed-solvent systems support this conclusion: a barrier of 24.7 kcal mol^–1^ was reported under neutral conditions, compared with 20.4 kcal mol^–1^ under acidic conditions.^37^ Similar trends were observed in water–DMSO mixtures, where barriers of approximately 21–23 kcal mol^–1^ (neutral) and 18.8–20.1 kcal mol^–1^ (acidic) were obtained.^48^ However, the rate was also found to depend strongly on the concentration of acids.^44–46,48^ For example, at sufficiently low acid concentrations, mutarotation can proceed more slowly under acidic conditions than it does in pure water. In contrast, no comparable effect is reported for basic media, where the reaction remains consistently faster over the concentration ranges studied.^44–46^ Interestingly, computational studies also find a higher barrier in acidic (20–25 kcal mol^–1^)^42,49,50^ than in neutral conditions (10–16 kcal mol^–1^),^50,51^ in line with the observed low-acid-concentration behavior.

On the basis of these kinetic results, mutarotation in solution appears to require both a proton donor (HA) and a proton acceptor (B), consistent with a general acid–base catalytic mechanism (Figure 2).^37,52^ Consequently, the ring-opening of d-glucose is commonly described as a three-step sequence: (i) deprotonation of the anomeric hydroxyl (O_1_–H_1_), (ii) protonation of the ring oxygen (O_5_), and (iii) cleavage of the C_1_–O_5_ bond. Although it is widely accepted that cleavage occurs at the C_1_–O_5_ bond, the identity and relative timing of the associated proton-transfer events remain unclear. This uncertainty arises because, in solution, proton shuttling can proceed through multiple pathways. By contrast, in the gas phase the proton transfer is intramolecular, which substantially simplifies the mechanism.^53,54^

**Figure 2:**
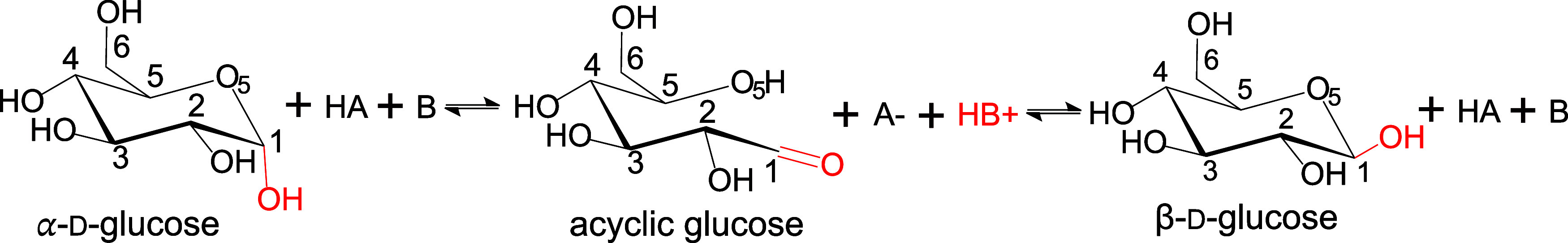
A general representation of the acid–base mechanism of d-glucose mutarotation. HA and B represents acid/proton donor and base/proton acceptor, respectively. In the absence of added acid or base, A=B=H_2_O.

Because the identities of HA and B can influence the sequence of steps (i)–(iii) outlined above, mutarotation is typically described by three pH-dependent pathways. Under acidic conditions (pH 1–4), HA predominates and protonation of O_5_ is expected to occur first, promoting cleavage of the C_1_–O_5_ bond; subsequent deprotonation of O_1_–H_1_ may then be assisted by water. Under basic conditions (pH 8–12), B dominates and initially abstract the O_1_–H_1_ proton, after which ring opening proceeds and water can protonate O_5_. In the intermediate pH range (4.5–6), water is proposed to facilitate both proton-transfer steps through hydrogen-bonded networks, but the sequence of these transfers has not been firmly established.^32,34–37,45^ Accordingly, two mechanistic scenarios have been put forward: stepwise versus concerted pathways.^34,36,37^

Within a stepwise framework, protonation of O_5_ could occur rapidly and reversibly, followed by a slower, rate-determining deprotonation of O_1_–H_1_ that produces the acyclic species. Alternatively, the initial proton-transfer event itself may be rate limiting, with subsequent structural reorganization occurring quickly. Pedersen proposed that protonation and deprotonation occur sequentially via discrete conjugate-acid or conjugate-base intermediates.^33^ More recently, Lewis and co-workers integrated kinetic isotope effect data with quantum calculations and found support for a stepwise mechanism in which a single water molecule plays the dominant role at the transition state.^55^ Consistent with this picture, Płaziński et al. used dynamical simulations and likewise identified a stepwise pathway featuring a single water molecule as the most favorable.^51^

In contrast, a concerted mechanism posits that both proton transfers occur simultaneously via a cyclic, multicenter transition state. Lowry and co-workers first proposed such a pathway, suggesting a trimolecular process involving the substrate, HA, and B, in which proton donation and proton abstraction occur simultaneously.^31,56^ Swain and Brown later reported kinetic observations consistent with a concerted route through a cyclic intermediate, including third-order behavior in inert solvents when both HA and B were present.^32,34,57^ They also showed that polyfunctional catalysts bearing two or more functional groups can simultaneously protonate O_5_ while deprotonating O_1_–H_1_. Subsequent experiments have further supported the plausibility of concerted pathways and invoked cyclic transition states.^7,35,36,48^ These ideas prompted the hypothesis that small, hydrogen-bonded clusters of water molecules might function as polyfunctional catalysts for proton transfer. However, experimental evidence for concerted transition states involving multiple water molecules remains indirect.^36,48^

The role of water molecules in d-glucose mutarotation has also been explored theoretically. Early quantum-mechanical studies proposed that multiple water molecules could participate in the water-catalyzed ring-opening step, with the lowest activation barriers predicted for pathways involving two or three waters.^58^ These interpretations were later challenged because they were based on static DFT calculations that did not explicitly account for solvent structure or dynamical effects.^54^

More recent dynamics simulations have been reported for mutarotation;^42,49,50^ however, they did not resolve either the number of water involved in catalysis or whether proton transfer occurs in a concerted or stepwise fashion. To address this gap, we examine the ring-opening reactions of both α- and β-d-glucose in aqueous solution, aiming to clarify the mechanism and to identify how many water molecules participate directly in catalysis. A key component of this work is a direct comparison between a stepwise pathway where O_5_ is protonated first and a potential concerted pathway, which has received limited attention in previous computational studies. To this end, we employ ab initio molecular dynamics (AIMD) combined with metadynamics, enabling us to capture solvent fluctuations alongside bond-making and bond-breaking events. From the resulting trajectories, we reconstruct free-energy surfaces (FES) to extract activation barriers and characterize the associated transition states (TS). Our findings show that a three-water intermediate is feasible and proceeds through a concerted proton-transfer mechanism.

## Results and Discussion

Here, we examine two plausible ring-opening mechanisms for d-glucose under water-only conditions, in which water functions as both proton donor and proton acceptor. The first is the widely accepted stepwise route, where O_5_ is protonated first. This scenario is analogous to ring opening under acidic conditions at low H_3_O^+^ concentration: proton transfer from H_3_O^+^ to O_5_ leaves only neutral water molecules in the immediate vicinity of d-glucose. The second is the mechanism that arises under neutral conditions when no prior mechanistic assumptions are imposed, thereby allowing for the possibility of a concerted ring-opening process.

Using AIMD simulations combined with metadynamics, we computed FESs as functions of three collective variables (CVs, Figure S1) for both the stepwise and concerted pathways, considering both α-d-glucose and β-d-glucose (Figure 3). Convergence of these simulations is shown in Figures S2–S5. Our d-glucose-water radial distribution functions (Figures S6–S7 and Table S1) also show that local waters are similar in all our simulations. This is further substantiated by the comparable number of d-glucose-water hydrogen bonds in our simulations (Table S2).

**Figure 3:**
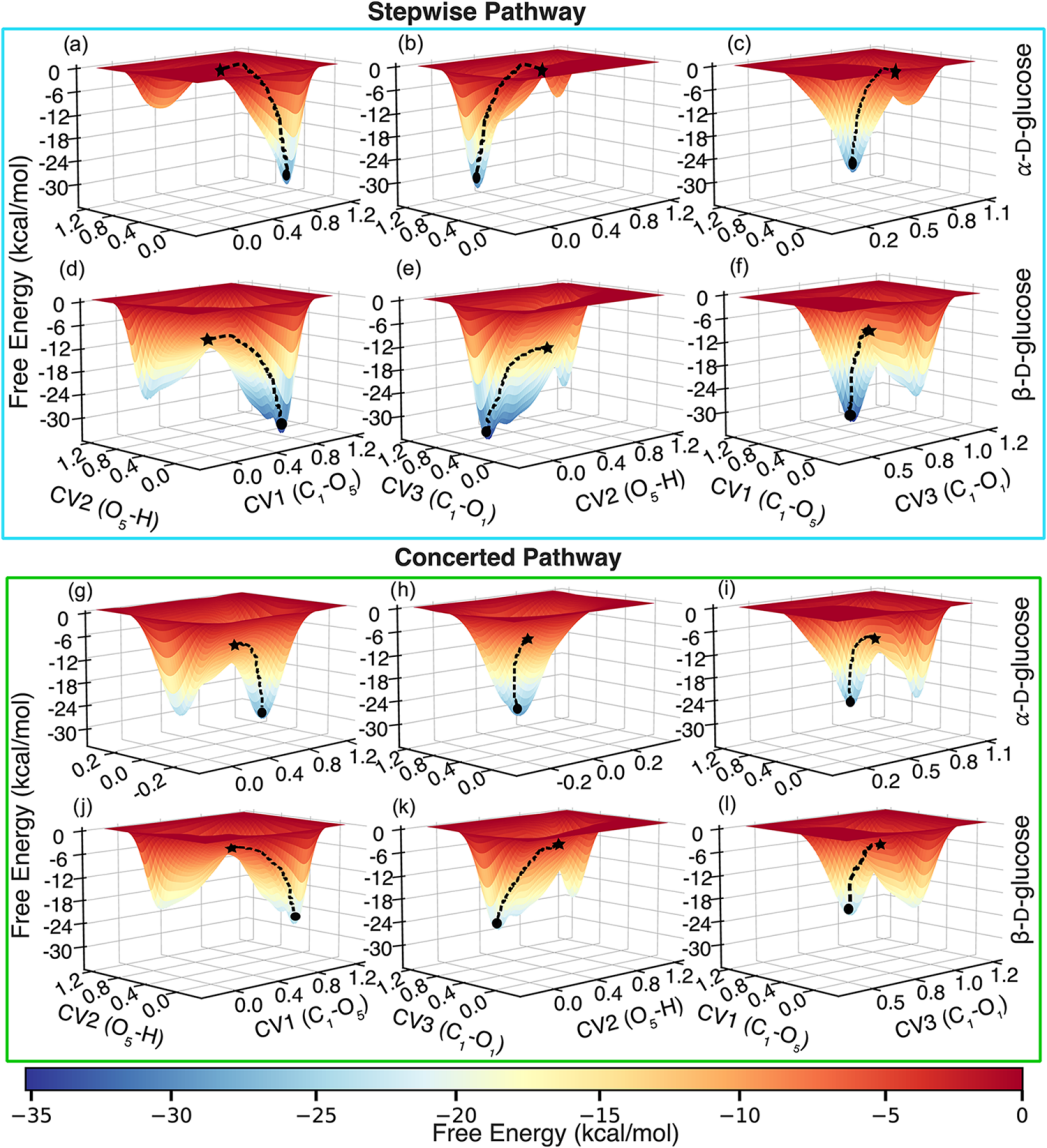
3D free energy surfaces for the ring-opening reaction for stepwise pathway (cyan box) and concerted pathway (green box) of α-d-glucose (a-c and g-i) and β-d-glucose (d-f and j-l): (a) CV2 (O_5_–H) vs CV1 (C_1_–O_5_) (b) CV3 (C_1_–O_1_) vs CV2 (O_5_–H) (c) CV1 (C_1_–O_5_) vs CV3 (C_1_–O_1_). The filled circle represents the reactant cyclic state and the star is the transition state.

In every case in Figure 3, the cyclic and acyclic forms of d-glucose appear as the only separate minima on the FES. The cyclic basin of α-d-glucose revolves around CV1 = 0.86 (1.48 Å), CV2 = 0.02 (2.86 Å), and CV3 = 0.87 (1.45 Å) while that of β-d-glucose is around CV1 = 0.85 (1.51 Å), CV2 = 0.04 (2.60 Å), and CV3 = 0.88 (1.43 Å).

For the stepwise pathway, we observe the acyclic basin of α-d-glucose around CV1 = 0.26 (2.38 Å), CV2 = 0.92 (1.00 Å), and CV3 = 0.94 (1.26 Å). This differs significantly from the acyclic basin of β-d-glucose for that same pathway: CV1 = 0.11 (2.80 Å), CV2 = 0.91 (1.03 Å), and CV3 = 0.95 (1.22 Å). Similarly, we see the minimum of the acyclic basin for α-d-glucose in the concerted pathway at CV1 = 0.10 (2.90 Å), CV2 = 0.00 (5.96 Å), and CV3 = 0.95 (1.23 Å) while it is located at CV1 = 0.17 (2.61 Å), CV2 = 0.91 (1.02 Å), and CV3 = 0.94 (1.25 Å) for β-d-glucose. The differences in the position of the acyclic basin suggests that the ring opens through different mechanisms, involving TS of differing geometry.

We then determine the minimum-energy pathways (MEPs) that connect the cyclic basin to the TS in each case. We extract the associated free-energy barriers and present the results in Table 1.

**Table 1:** Free Energy Barriers in kcal mol^–1^ for the Ring-Opening of α- and β-d-Glucose for the Stepwise and Concerted Mechanisms^a^

in kcal mol^–1^	Stepwise	Concerted
α-d-glucose	28.1	15.4
β-d-glucose	20.3	18.0

a The barriers were computed from a minimum energy paths on the reconstructed FES presented in Figure 3, as detailed in Supporting Information.

The stepwise pathway yields the largest barriers, spanning 20–28 kcal mol^–1^, whereas the concerted pathway is consistently lower, with barriers in the 15–18 kcal mol^–1^ range. The stepwise barriers are in line with experimental activation barriers reported at 21–25 kcal mol^–1^,^37,48^ and they also match prior theoretical estimates for the stepwise route, including 20–25 kcal mol^–1^ for the β-anomer^42,49^ and ∼19 kcal mol^–1^ for the α-anomer.^49^ Previous theoretical results also lists 10–15 kcal mol^–1^ for the concerted pathway.^50,51^ To better understand the differences in FES and barrier heights, we explore the dynamics of each reaction in details.

### Stepwise Ring-Opening of d-Glucose

Here, the metadynamics simulations were initiated with a proton bound to O_5_ for both cyclic anomers of d-glucose, as in previous studies.^42,49^ Figure 4 shows the variation in the bond distances as a function of simulation time (ps) for α-d-glucose. A similar profile is shown in Figure S8 for β-d-glucose.

**Figure 4:**
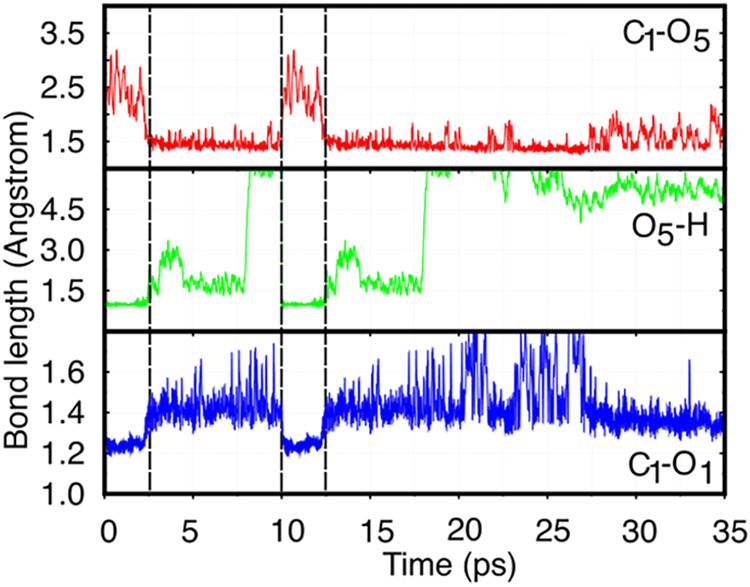
Variation in bond lengths during the metadynamics simulations (in ps) for cyclic α-d-glucose conversion to acyclic for stepwise ring-opening. C_1_–O_5_ (CV1) is red; O_5_–H (CV2) is green; C_1_–O_1_ (CV3) is blue.

Figure 4 shows that the C_1_–O_5_ bond starts elongated at about 2.5 Å, indicating that bond cleavage occurs immediately. Meanwhile, the C_1_–O_1_ bond length is about 1.2 Å, consistent with the double-bond character of the acyclic intermediate. The opening of the ring immediately following the protonation of O_5_ (time 0 in our simulations) has been observed elsewhere.^49,50^ At 2.5 ps, a sudden increase in the O_5_–H distance indicates proton transfer from O_5_ to a nearby water molecule. This event promotes ring closure and restoration of the cyclic structure, with the C_1_–O_5_ bond shortening to about 1.5 Å and the C_1_–O_1_ bond lengthening to approximately 1.5 Å, consistent with single-bond character. The proton subsequently remains in the solvent for about 7.5 ps before returning to O_5_ at 10.0 ps, thereby initiating a second ring-opening event. This transient opening is followed by ring closure and deprotonation of O_5_ at approximately 12.5 ps, after which the ring remains closed for the rest of the trajectory.

Interestingly, ring opening in β-d-glucose proceeds differently. Over the first 15 ps, the C_1_–O_5_ and C_1_–O_1_ bond lengths remain approximately constant at 1.5 Å, whereas the O_5_–H bond length changes markedly, from 1.5 Å to 7.5 Å and then to 4.5 Å, indicating proton shuttling between O_5_ and neighboring water molecules. At 35 ps, the O_5_–H distance returns to about 1.6 Å, consistent with a hydronium-like water carrying the excess proton in the immediate vicinity of O_5_. The proton becomes firmly associated with O_5_ at 115 ps, forming an O_5_–H bond of approximately 1.00 Å. This event precedes elongation and eventual cleavage of the C_1_–O_5_ bond at 115 ps, leading to ring opening. This exchange of proton between O_5_ and water is consistent with earlier studies of β-d-glucose ring opening,^42^ but differs from more recent work,^49,50^ reporting immediate and complete cleavage of the C_1_–O_5_ bond upon initial protonation in both anomers. Note also that, although the C_1_–O_5_ bond fully dissociates at 115 ps and remains broken for the rest of the simulation, transient ring-opening and ring-closing events occur during the first 110 ps, as indicated by fluctuations in the C_1_–O_5_ bond length between 1.40 and 2.40 Å.

Finally, Figure 5 presents the TS structures for both anomers (snapshots with nearby water molecules are shown in Figure S12). For α-d-glucose, the TS features a proton directly bound to O_5_. In contrast, the TS of the β-anomer contains a water molecule oriented to transfer its proton to O_5_. These structural differences are consistent with the simulation dynamics: for the α-anomer, ring opening follows almost immediately upon protonation of O_5_, whereas for the β-anomer, the proton first dissociates from O_5_ to a nearby water molecule and only later returns to O_5_ prior to ring opening. The transfer of the proton to water and its subsequent return to O_5_ support our initial assumption that O_5_ is protonated in the starting structure and further indicate that protonation must precede cleavage of the C_1_–O_5_ bond, in agreement with previous studies.^42,48^ However, the substantially higher activation barrier for the α-anomer relative to the β-anomer (Table 1) suggests that water-assisted cooperation is essential to the stepwise mechanism.

**Figure 5:**
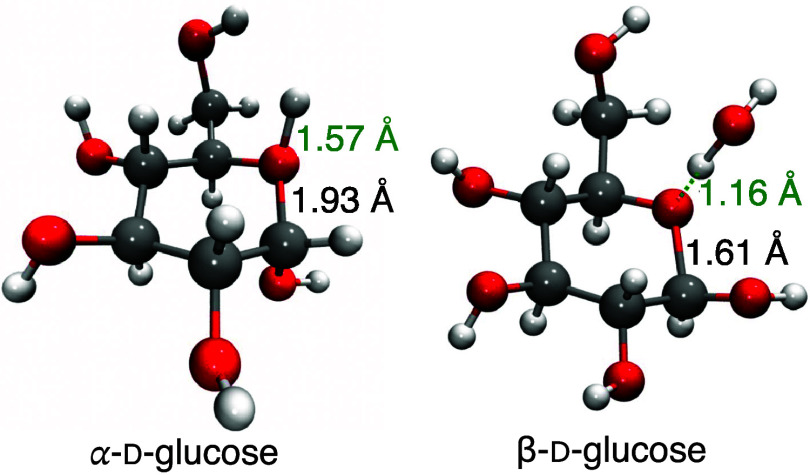
Transition state structures of α- and β-d-glucose for the stepwise pathway. C_1_–O_5_ bond and O_5_–H bond distances are labeled in black and green, respectively.

Having established the important role of water in both the TS structure and the overall ring-opening mechanism, we next investigate the ring opening of α- and β-d-glucose under neutral conditions without imposing any prior mechanistic assumptions, thus allowing for the possibility of a truly concerted ring-opening process.

### Concerted Ring-Opening of d-Glucose

Here, we started our metadynamics simulations with only neutral water in the vicinity of the d-glucose anomers. The time evolution of C_1_–O_5_ (CV1), O_5_–H (CV2), and C_1_–O_1_ (CV3) bond distances are shown in Figures S9–10 for the α and β anomers, respectively.

Over the first 25 ps of the α-d-glucose trajectory, the only change observed is a reorganization of the surrounding water molecules. This process features gradual formation of an hydrogen-bond network linking water to the reactive region of d-glucose. After 25 ps, the C_1_–O_5_ bond gradually elongates from 1.50 Å to more than 3.5 Å. By contrast, the O_5_–H and C_1_–O_1_ distances decrease to 1.0 and 1.2 Å, respectively, and remain at those values for the remainder of the simulation. At 25.5 ps, complete ring opening is observed: O_5_ becomes fully protonated, with an O_5_–H bond length of 1.03 Å, while the C_1_–O_5_ distance extends to 2.52 Å.

Analysis of the metadynamics simulation beyond the chosen CVs reveals that the ring-opening event described above is preceded by deprotonation of the hydroxyl group at position 1 (OH_1_) by a water molecule (Figure 6a). The released proton is subsequently transferred through a hydrogen-bond network formed by three neighboring water molecules, which effectively shuttles the proton from OH_1_ to O_5_ (Figure 6c–e). To the best of our knowledge, this is the first direct MD observation of a three-water proton relay during d-glucose ring opening. Previous static DFT studies had suggested that three water molecules might lower the ring-opening barrier,^58^ whereas later work combining DFT and MD found that, although two water molecules could initially mediate the process, the more favorable pathway was a stepwise single-water mechanism.^51^ However, our simulations clearly show that, in the absence of any a priori mechanistic assumptions, a three-water-mediated pathway is preferred. Importantly, β-d-glucose follows a similar mechanism (Figure S11). As in the α anomer, the first 27 ps involves mainly water reorganization and the establishment of a hydrogen-bond network around the reactive region. At 27.50 ps, the O_5_–H and C_1_–O_1_ distances contract to 1.2 Å, while the C_1_–O_5_ distance increases from 1.45 Å to 2.2 Å. This sequence is again preceded by deprotonation of OH_1_ by a neighboring water molecule, after which the released proton is transferred to O_5_ through a three-water hydrogen-bond relay. The subsequent elongation of the C_1_–O_5_ bond to 2.67 Å at 27 ps and 3.21 Å at 32 ps marks the formation of acyclic β-d-glucose, while the O_5_–H and C_1_–O_1_ bond lengths decrease to 0.96 Å and 1.20 Å, respectively.

**Figure 6:**
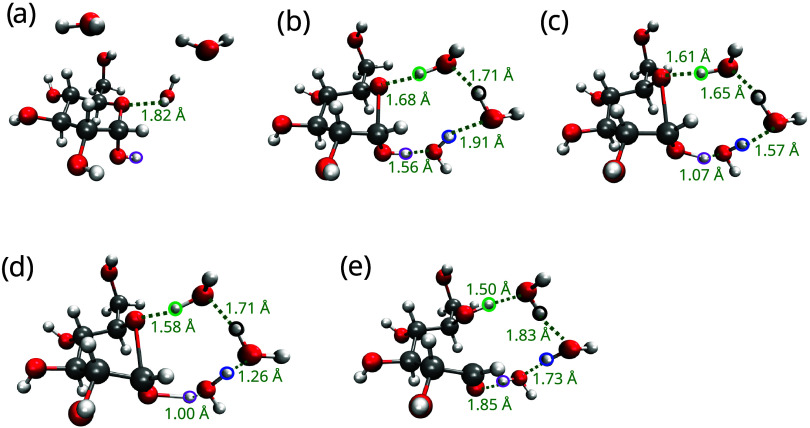
Representative snapshot illustrating the proton relay process involved in the concerted ring-opening reaction of α-d-glucose at (a) 0 (b) 25.15 ps (c) 25.25 ps (d) 25.35 ps (e) 25.45 ps. Color circles (purple, blue, black and green) highlight the protons involved.

Figure 7 shows the TS structures obtained from our simulations. In both anomers, the TS features a slight elongation of the C_1_–O_5_ bond and shortening of the O_5_–H and C_1_–O_1_ bonds. Notably, each TS structure is stabilized by the three-water hydrogen-bond network that shuttle the proton from O_1_ to O_5_.

**Figure 7:**
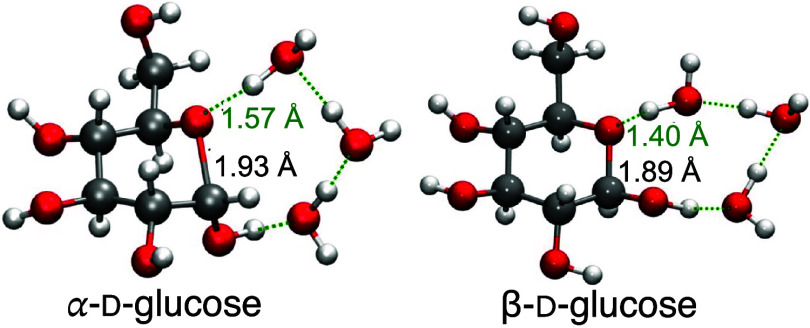
Transition state structures of α- and β-d-glucose for concerted pathway. C_1_–O_5_ bond and O_5_–H bond distances are labeled in black and green, respectively.

This geometry supports the feasibility of a multiwater hydrogen-bonded arrangement and addresses earlier concerns about the ability of such configurations to participate in the ring-opening transition state.^54^ Importantly, complete cleavage of the C_1_–O_5_ bond takes place only after the proton is fully transferred to O_5_. The sequence of structural changes therefore suggests an asynchronous but concerted mechanism, in which the O_1_–H_1_ proton is released early and relayed through the three-water network to O_5_. This interpretation is in line with classical views of concerted acid–base catalysis^30,35,36,48,59^ and with established models of coupled proton-transfer processes.^34,60,61^ Notably, Gandour and co-workers^60^ showed that bifunctional catalytic systems with coupled proton motions are most stable in a cyclic “supermolecular” hydrogen-bonding arrangement, emphasizing the energetic benefit of cooperative hydrogen bonding.

Interestingly, the calculated barrier for ring opening of α-d-glucose is about 2.5 kcal/mol lower than that of β-d-glucose (Table 1). Inspection of the TS structures in Figure 7 suggests that this difference likely reflects a more strained hydrogen-bond network in the β anomer relative to the α anomer. This finding is consistent with the fact that no significant rate difference has been measured between α- and β-d-glucose, indicating that the reaction environment exerts a stronger influence than the identity of the anomer.^13,14,45,47^

Finally, we observe that 1.0 ps after the ring opening of β-d-glucose, the system recloses, with the C_1_–O_5_ bond contracting to 1.85 Å and the C_1_–O_1_ and O_5_–H distances increasing simultaneously to 1.2 Å. Examination of the extended trajectories reveals a dynamic succession of ring-opening and reclosure events, leading to accessible anomeric forms of d-glucose. At around 34.6 ps, for instance, an α-like anomeric structure is formed, marked by C_1_–O_5_ and C_1_–O_1_ distances of 1.56 Å and 1.36 Å, respectively, and a downward orientation of O_1_. This state, however, is not protonated at O_1_ and persists for only about 4.3 ps before returning to the acyclic form. Multiple transitions between the α and β anomers via the acyclic intermediate are observed during the simulation, emphasizing the dynamic equilibrium among these states. This result agrees with previous experimental findings that mutarotation proceeds through ring–chain tautomerism, in which the acyclic form serves as a transient intermediate.^7,62^ No significant upward or downward reorientation of the O_1_ group was observed in the stepwise pathway.

To further quantify the active role of water in ring opening, we calculated the ring strain energy of d-glucose in each case (Table S3). We found that the concerted pathway exhibited higher ring strain energies for both anomers, suggesting greater stabilization of the cyclic state relative to the acyclic intermediate. The lower energy barriers (in water) reported for the concerted pathway despite the higher ring strain energies (in vacuum), reinforces that water plays a greater stabilizing role than in the stepwise pathway. The enhanced solvent assistance, rather than ring strain effects, appears to be the dominant factor lowering the barrier in this mechanism.

## Conclusion

In this paper, we employed AIMD combined with metadynamics in explicit solvent to examine two possible mechanisms for the ring opening of d-glucose: a stepwise pathway in which O_5_ is protonated first, and an unrestricted pathway that allows for a concerted mechanism.

Our simulations show that although the C_1_–O_5_ bond cleaves only after protonation of O_5_ in the stepwise pathway, the TS can be stabilized by surrounding water, lowering the barrier height. Nevertheless, we find that the kinetically preferred route is the asynchronous concerted pathway. Our results support a concerted proton-transfer mechanism characterized by a TS in which three water molecules bridge O_1_–H_1_ and O_5_ for both α- and β-d-glucose. This picture aligns with early experimental proposals invoking multiwater participation,^36,48^ yet stands in clear contrast to prior computational studies that favored a stepwise pathway involving a single water molecule without direct bridging between O_1_–H_1_ and O_5_.^51,55^

Overall, our AIMD simulations provide the first direct evidence that ring opening in d-glucose is mediated by a cyclic, three-water hydrogen-bonded network. More broadly, the identification of a solvent-assisted proton relay emphasizes the role of hydrogen-bond networks in lowering activation barriers and modulating reaction rates, as widely recognized in enzymatic catalysis and related biological processes.^63–70^ The presence of such a three-water relay in d-glucose mutarotation carries important implications for carbohydrate solution chemistry and for the mechanistic understanding of early glycation events. In particular, conversion to the reactive open-chain aldehyde form is a prerequisite for Schiff base formation with protein amino groups, a step widely considered rate-limiting in the Maillard reaction.

## Computational Details

### Starting Geometries

α- and β-d-glucose in the ^4^C_1_ ring conformation were each placed in the center of a 15.264 × 15.264 × 15.264 Å^3^ box. 99 water molecules were added from a pre-equilibrated water box using the gromacs solvate algorithm^71,72^ to reach a density of 1 g/mL. Each structure was then minimized with the steepest descent, using the AMOEBA polarizable force field,^73,74^ within tinker9.^75^ The minimized structures were then used to start a 300 ns MD simulation under constant number of particles (321 atoms), pressure (1 atm) and temperature (300 K). The Nose-Hoover thermostat and barostat were employed to maintain the temperature and pressure.^76,77^

### Ab Initio Metadynamics

AIMD simulations coupled with well-tempered metadynamics were performed using the QUICKSTEP module of the CP2K package.^78^ Atomic forces were calculated using DFT with the BLYP exchange-correlation functional.^79,80^ The choice of BLYP functional is based on previous work that showed its reliability in describing d-glucose and other carbohydrate processes in aqueous solution.^42,81,82^ Kohn–Sham orbitals were represented using a hybrid Gaussian/plane-wave basis set approach.^83^ Double-zeta valence polarized (DZVP) basis sets^84^ and Goedecker-Teter-Hutter pseudopotentials^85^ were employed for all atoms, with the auxiliary plane-wave basis expanded up to a cutoff of 400 Ry. Dispersion interactions were accounted for using the Grimme D3 correction.^86^ A convergence criterion of 10^–5^ hartree was imposed on the ground-state energy. All simulations were conducted in the canonical (NVT) ensemble at 300 K, using a Nosé-Hoover thermostat^76,77^ with a time constant of 1 ps.

The last frame from the classical MD simulations was used to initialize the AIMD simulations. For the concerted pathway, the system was used without modification from the classical MD, comprising 321 atoms, including d-glucose and 99 water molecules. For the stepwise pathway, the initial structure was modified by protonating the O_5_ atom, consistent with previous studies,^42,49^ and introducing a Cl^–^ counterion to maintain charge neutrality, resulting in a system of 323 atoms. All AIMD simulations were conducted in a cubic periodic box with dimensions of 14.866 × 14.866 × 14.866 Å^3^, employing periodic boundary conditions in all three directions. The reaction was described using coordination numbers as CVs. Specifically, three CVs, CV1, CV2 and CV3, were used to track the structural changes along the reaction pathway, as illustrated in Figure S1. CV1 is the coordination number between C_1_ and O_5_, CV2 the coordination number between O_5_ and a solvent hydrogen atom (H*), and CV3 the coordination number between C_1_ and O_1_. In each case, the coordination number was calculated according to

1 $$\mathrm{CN}=\frac{1-{\left(\frac{d}{d_{0}}\right)}^{6}}{1-{\left(\frac{d}{d_{0}}\right)}^{12}}$$

where *d* is the interatomic distance and *d*_0_ is a reference distance. Based on previous studies,^42,49^ *d*_0_ = 1.5 Å and 2.0 Å for C–O and O–H bonds, respectively.

In the metadynamics simulations, we deposited Gaussian bias potentials with a height of 0.001 Ha and width of 0.08 au every 50 steps, with a time step of 0.5 fs. Free energy surfaces were reconstructed using the graph module of CP2K, which reads the positions, heights, and widths of the deposited Gaussian to compute the free energy landscape. MEPs were subsequently determined using the zero-temperature string method of Maragliano et al.^87^ implemented in Python, employing 16 images and 10,000 optimization steps. The average number of hydrogen bonds surrounding the d-glucose oxygen atoms was quantified using an O–H distance cutoff of 3.0 Å and an O–H···O angle criterion of 30°.

We also compute the ring strain energy (in kcal/mol) as the excess energy between the cyclic and the acyclic configuration of d-glucose in absence of water. Here, we used the ten frames closest to the corresponding free-energy minima to obtain statistically representative low-energy conformations. Water molecules were subsequently removed from the extracted structures. We calculated single-point energies for the dehydrated cyclic and acyclic structures, and evaluated the ring strain energy as

2 $$\Delta E_{\left(\mathrm{ringstrainenergy}\right)}=E_{\mathrm{acyclic}}-E_{\mathrm{cyclic}}$$

### Convergence Criteria

Convergence analysis is critical in enhanced sampling approaches, as insufficient sampling can lead to artificial bias or incomplete representation of the underlying energy landscape. In this study, convergence was assessed based on two criteria: (1) in the well-tempered metadynamics simulations, the Gaussian height decreased over time and reached a small value (ca. < 0.1 kcal/mol); (2) the stability of the FES profiles over extended simulation times. These checks confirm that the reconstructed FES accurately capture the thermodynamic features of the ring-opening process. Convergence plots are shown in Figures S2–S5.

## Supplementary Material



## Notes

Convergence plots of Gaussian heights and free energies; a discussion of radial distribution functions and hydrogen-bond count analysis; analyses of bond length variations; representative snapshots illustrating the proton relay process in the concerted pathway of α-d-glucose; and ring strain energy calculations (PDF)
