# Supplementary material for: Three Water Molecules Mediate Ring Opening of d‑Glucose in Aqueous Solutions
Source: ACS Phys Chem Au. 2026 Jun 9;6(4):869–77. doi: 10.1021/acsphyschemau.6c00055 (PMC13397440; doi:10.1021/acsphyschemau.6c00055)
Supplement: Supplementary file 1 [file pg6c00055_si_001.pdf]

Three Water Molecules Mediate Ring Opening of D-Glucose in Aqueous Solutions Supporting Information

Mawuli Deegbey† and Valerie Vaissier Welborn*,†

† *Department of Chemistry, Virginia Tech, Blacksburg, Virginia, USA 24061*

‡ *Macromolecules Innovation Institute, Virginia Tech, Blacksburg, Virginia, USA 24061*

E-mail: vwelborn@vt.edu

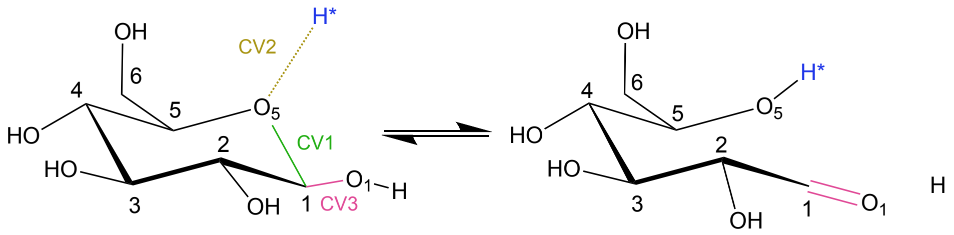

	CV1	CV2	CV3
Cyclic (Å)	0.85 (1.43 Å)	0.00 (2.09 Å)	0.88 (1.36 Å)
Acyclic (Å)	0.00 (2.50 Å)	0.94 (0.96 Å)	0.93 (1.20 Å)

Figure S1: CV employed in the well-tempered metadynamics simulations. CV1 is the CN between C_1 and O_5, which tracks the C_1–O_5 bond cleavage. CV2 is the CN between O_5 and a solvent hydrogen atom (H*), monitoring proton transfer events. CV3 corresponds to the CN between C_1 and O_1, tracking the formation of a double bond when in the open-chain form. The corresponding bond distances in Å associated with each CV are also indicated in parenthesis.

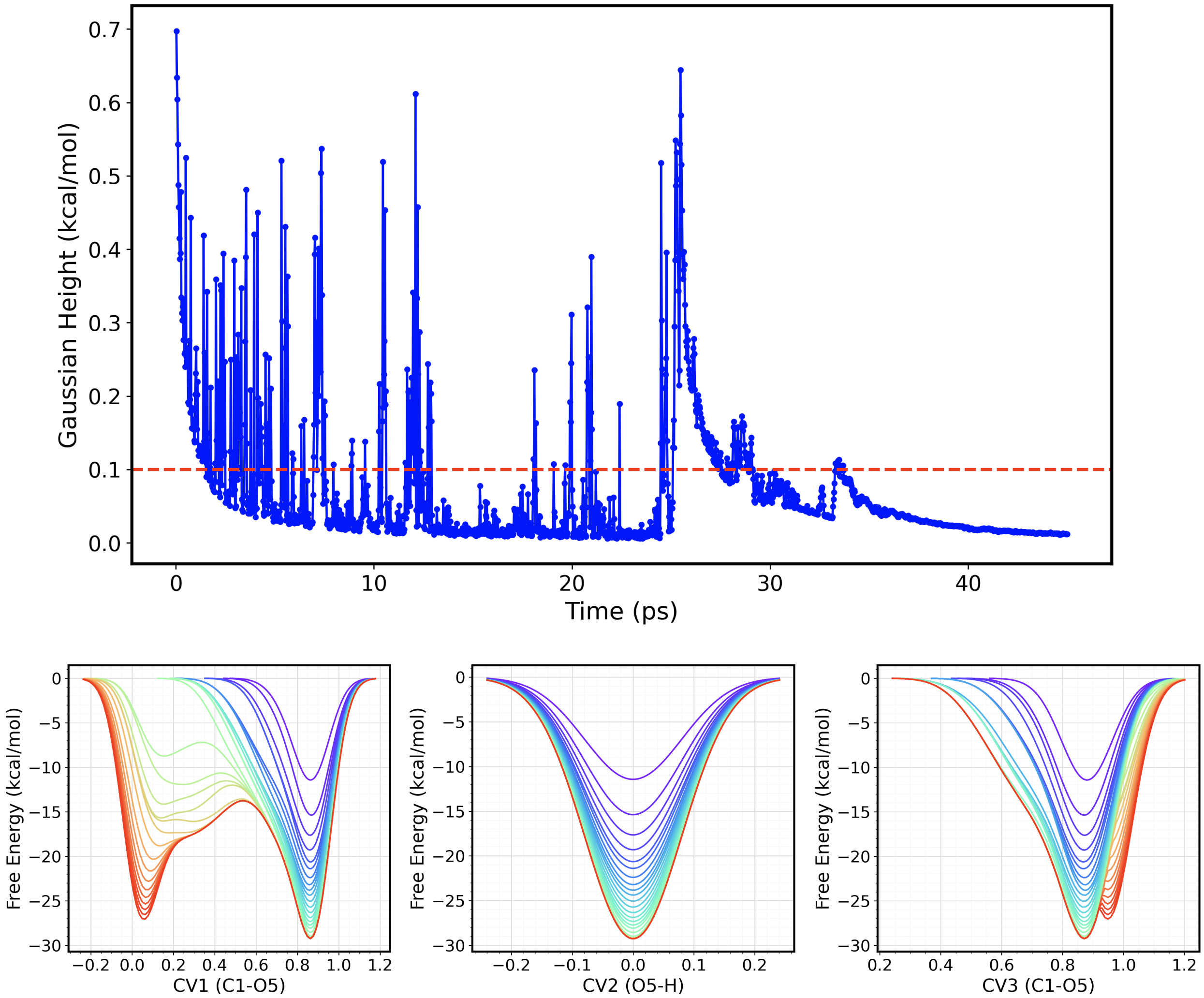

Figure S2: Convergences of the free energy simulation with metadynamics for free energy profile of the concerted pathway of α-D-glucose. Changes in Gaussian height (top) and energy convergence for CV1, CV2 and CV3 (bottom). A decrease in Gaussian height below 0.1 kcal/mol was employed as a criteria for simulation convergence

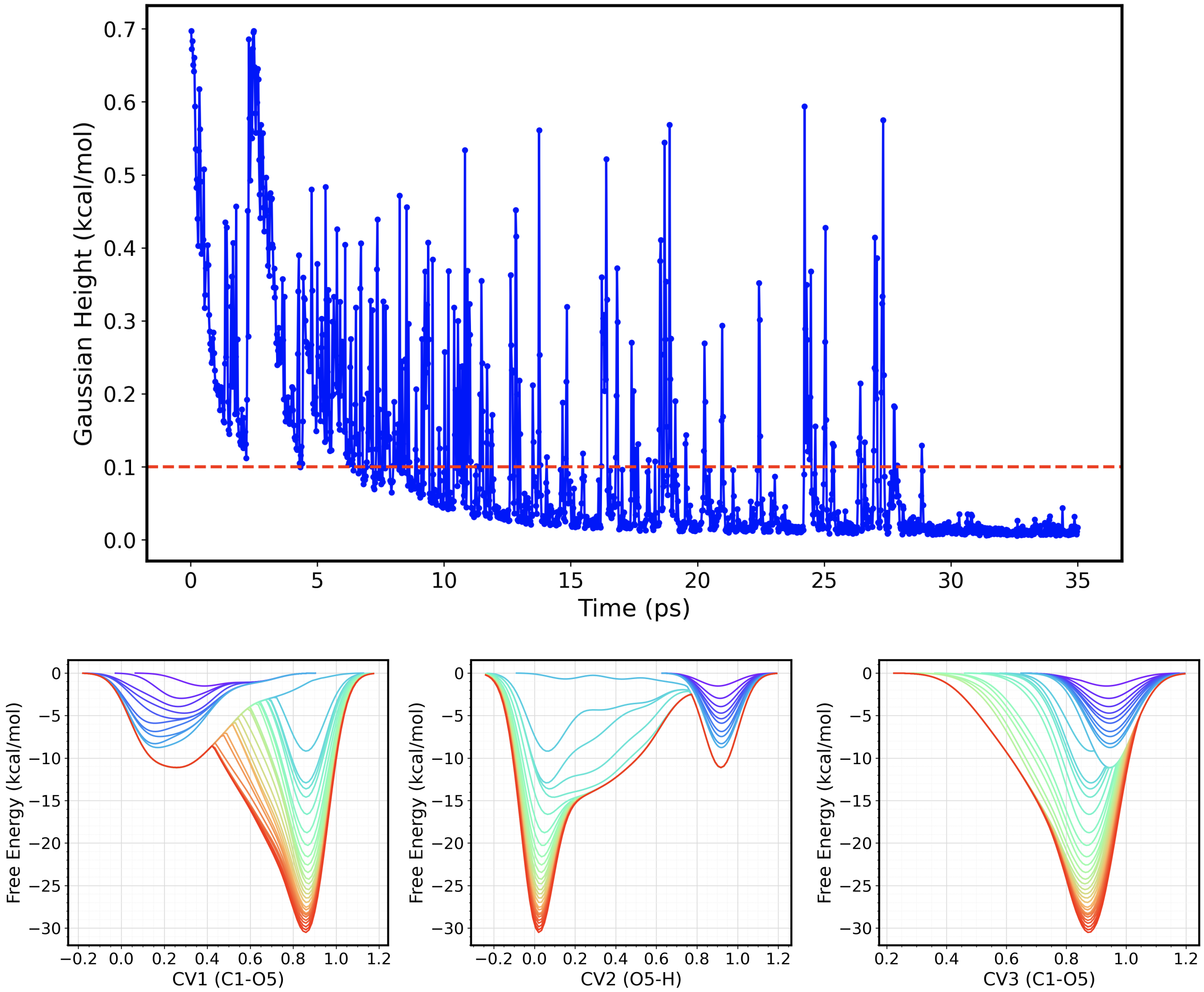

Figure S3: Convergences of the free energy simulation with metadynamics for free energy profile of the stepwise pathway of α-D-glucose. Changes in Gaussian height (top) and energy convergence for CV1, CV2 and CV3 (bottom). A decrease in Gaussian height below 0.1 kcal/mol was employed as a criteria for simulation convergence

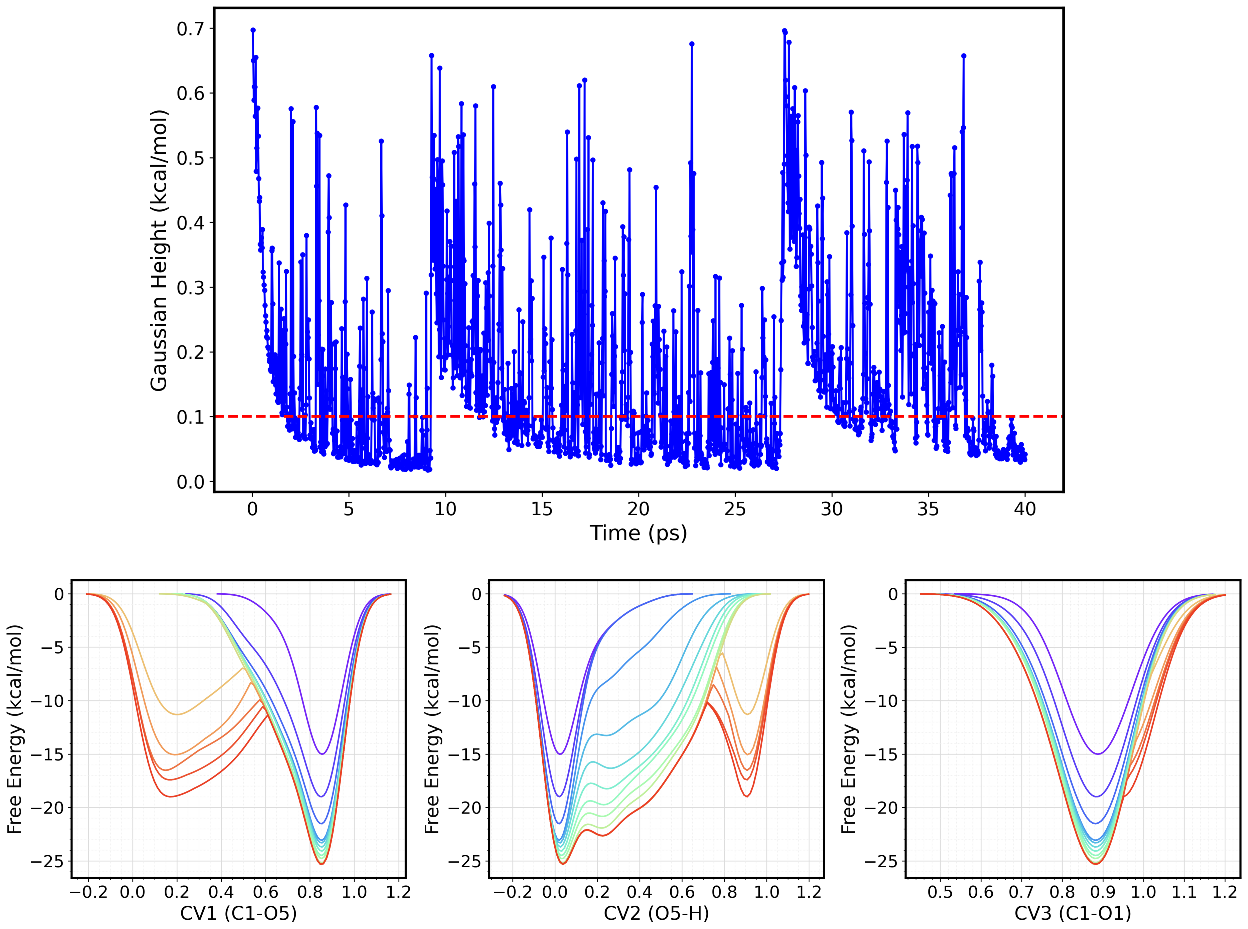

Figure S4: Convergences of the free energy simulation with metadynamics for free energy profile of the concerted pathway of β-D-glucose. Changes in Gaussian height (top) and energy convergence for CV1, CV2 and CV3 (bottom). A decrease in Gaussian height below 0.1 kcal/mol was employed as a criteria for simulation convergence

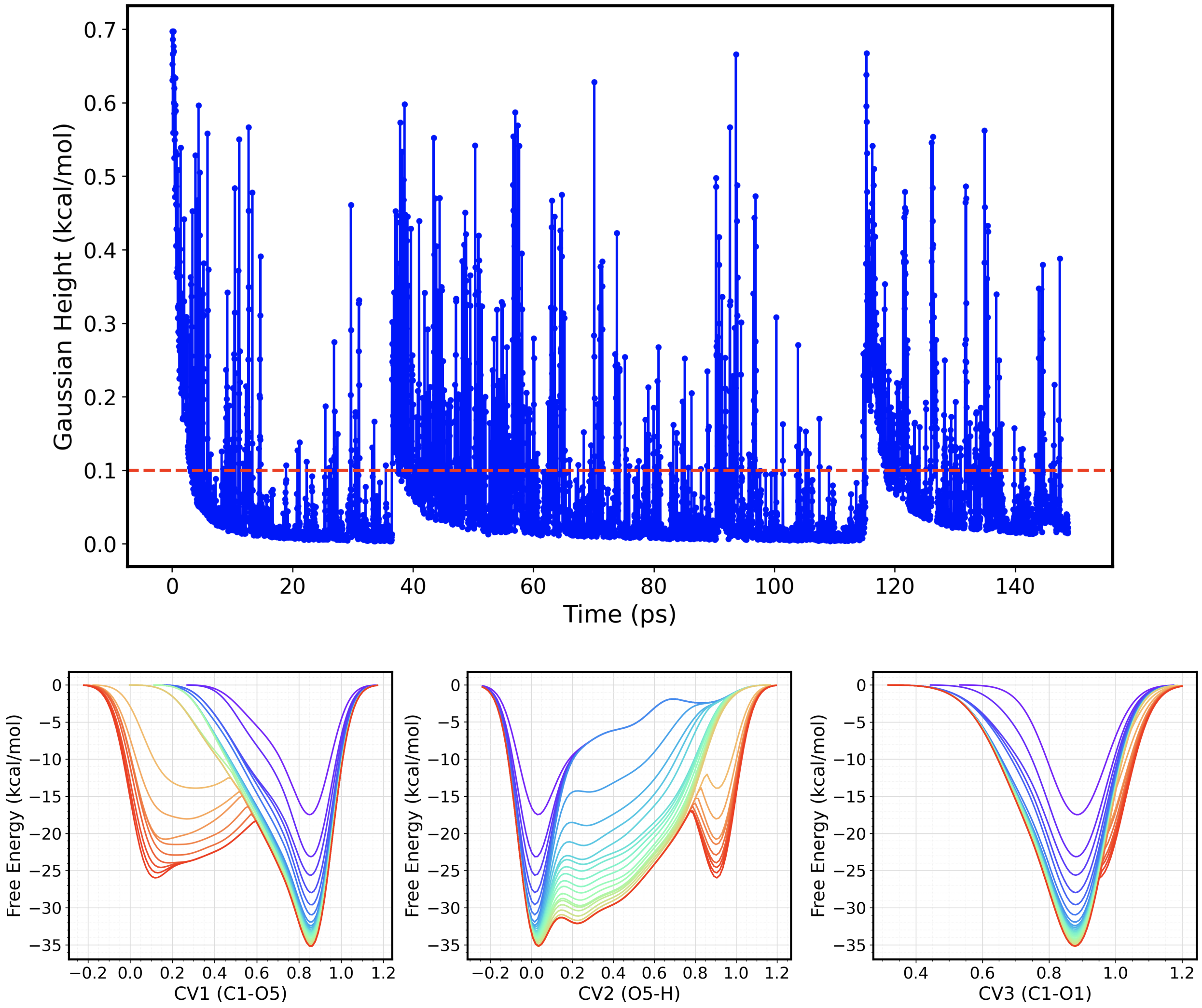

Figure S5: Convergences of the free energy simulation with metadynamics for free energy profile of the stepwise pathway of β-D-glucose. Changes in Gaussian height (top) and energy convergence for CV1, CV2 and CV3 (bottom). A decrease in Gaussian height below 0.1 kcal/mol was employed as a criteria for simulation convergence

Radial distribution functions

The radial distribution functions of glucose oxygen (Og) and water oxygen (Ow) atoms for the stepwise and concerted pathways are shown in Figures S6 and S7 for both α- and β-D-glucose, respectively. In both anomers, the radial distribution function for all glucose oxygen atoms and water oxygen atoms displays a first maximum at ∼2.8 Å, characteristic of hydrogen-bonded O-O interactions, followed by a well-defined first solvation shell extending to ∼3.4–4.0 Å. Integration of the first shell for O_1-O_6, yields coordination number (CN) of ∼4.1 for both anomers, suggesting that each glucose oxygen is solvated on average by approximately four water molecules. The total average CNs (see Table S1), were further decomposed into cyclic and acyclic configurations. For all oxygen atoms (O_1-O_6), we observe no significant difference in CNs between the cyclic and acyclic states. Further analysis of the oxygen atoms (O_1 and O_5) involved in the proton transfer, reveals a greater number of water molecules around O_1 (∼5) compared to O_5 (∼3).

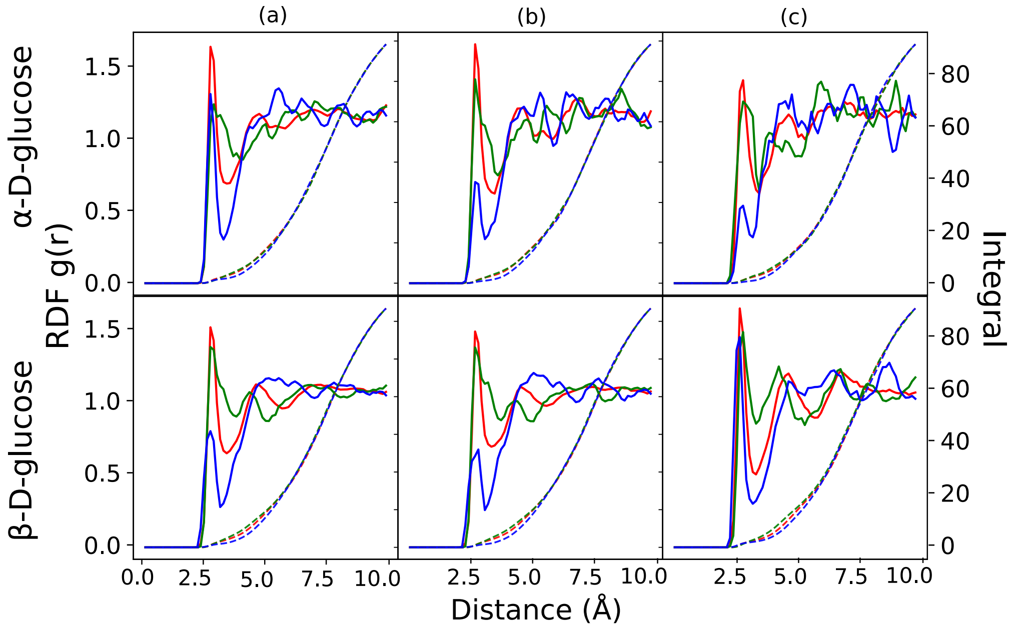

Figure S6: Radial distribution functions $g(r)_{og-ow}$ for D-glucose along the stepwise pathway: (a) combined cyclic and acyclic configurations (b) only cyclic state (c) only acyclic state. In each panel, the red line represents all glucose oxygen atoms (O_1–O_6) relative to water oxygen atoms (Ow), the green represents O_1–Ow, and the blue represents O_5–Ow.

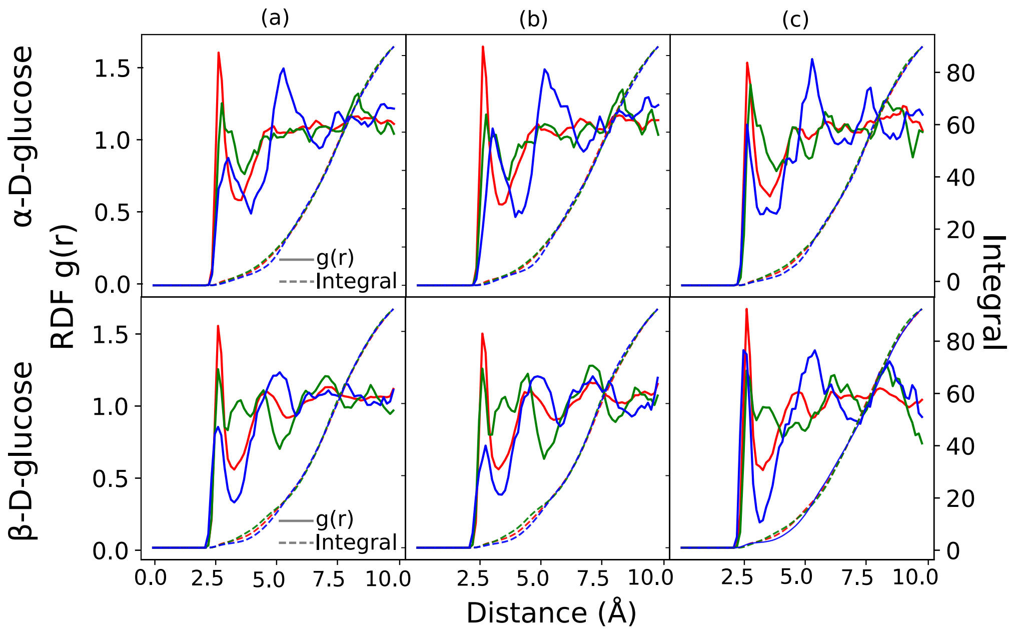

Figure S7: Radial distribution functions $g(r)_{og-ow}$ for D-glucose along the concerted pathway: (a) combined cyclic and acyclic configurations (b) only cyclic state (c) only acyclic state. In each panel, the red line represents all glucose oxygen atoms (O_1–O_6) relative to water oxygen atoms (Ow), the green represents O_1–Ow, and the blue represents O_5–Ow.

Table S1: Total average coordination numbers for α- and β-D-glucose for the stepwise (concerted) pathway based on a first minima at 3.8 Å

	Combined	Cyclic	Acyclic
	α-D-glucose		
O_1-O_6	4.2 (4.1)	4.0 (4.1)	4.1 (4.2)
O_1	4.7 (4.6)	4.4 (4.4)	4.8 (4.9)
O_5	2.8 (3.5)	2.5 (3.8)	3.0 (3.1)
	β-D-glucose		
O_1-O_6	4.1 (4.1)	4.3 (4.1)	3.8 (4.1)
O_1	5.2 (5.2)	5.2 (5.2)	5.0 (5.1)
O_5	2.7 (2.7)	2.6 (2.7)	3.0 (2.9)

Hydrogen Bond Count Analysis

The average number of hydrogen bonds surrounding the D-glucose oxygen atoms was quantified using an O–H distance cutoff of 3.0 Å and an O−H···O angle criterion of 30°. Our analysis reveals clear differences in solvent organization between the stepwise pathway and the concerted pathway. Overall, the two pathways exhibit comparable hydrogen-bonding patterns, with only subtle deviations in hydrogen bond counts at the acyclic O_1–O_6 positions. A focused examination

of the reactive centers, O_1 and O_5, shows a modest but consistent increase in hydrogen bond count for β-D-glucose, suggesting enhanced solvent stabilization at these sites during the reaction.

Table S2: Average number of hydrogen bonds for α- and β-D-glucose for the stepwise (concerted) pathway

	**Combined**	Cyclic	Acyclic
		α-D-glucose	
O1-O6	6.1 ± 1.6 (7.6 ± 1.6)	6.2 ± 1.5 (8.0 ± 1.5)	6.3 ± 1.5 (7.1 ± 1.5)
O1	1.0 ± 0.7 (0.8 ± 0.6)	1.1 ± 0.7 (0.8 ± 0.7)	0.9 ± 0.5 (0.7 ± 0.6)
O5	0.6 ± 0.6 (0.5 ± 0.6)	0.4 ± 0.5 (0.2 ± 0.4)	0.8 ± 0.6 (0.9 ± 0.5)
		β-D-glucose	
O1-O6	5.2 ± 2.0 (8.5 ± 1.6)	5.2 ± 2.0 (8.3 ± 1.6)	4.2 ± 1.2 (8.8 ± 1.5)
O1	0.4 ± 0.7 (1.1 ± 0.6)	0.4 ± 0.7 (1.2 ± 0.6)	0.0 ± 0.1 (1.0 ± 0.6)
O5	0.8 ± 0.6 (1.1 ± 0.6)	0.8 ± 0.6 (0.9 ± 0.6)	1.2 ± 0.6 (1.6 ± 0.5)

Bond Length Variation

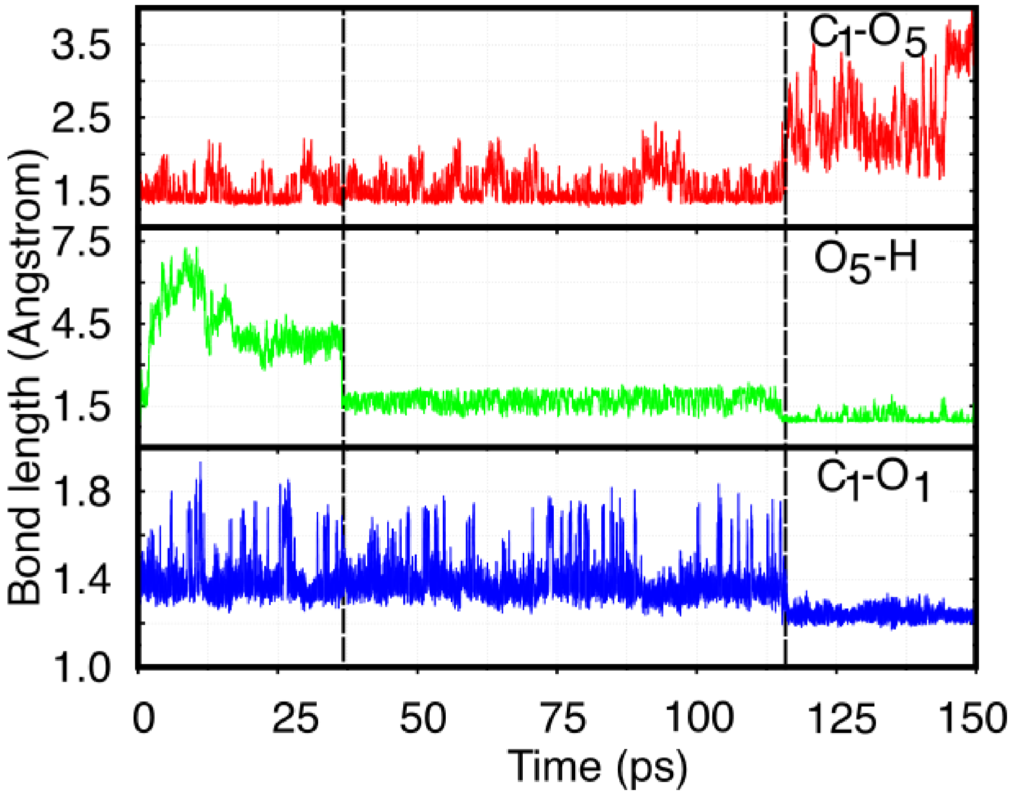

Figure S8: Variation in bond lengths during the metadynamics simulations (in ps) for cyclic β-D-glucose conversion to acyclic form for stepwise pathway. C1-O5 (CV1) is red, O5-H (CV2) is green, C1-O1 (CV3) is blue.

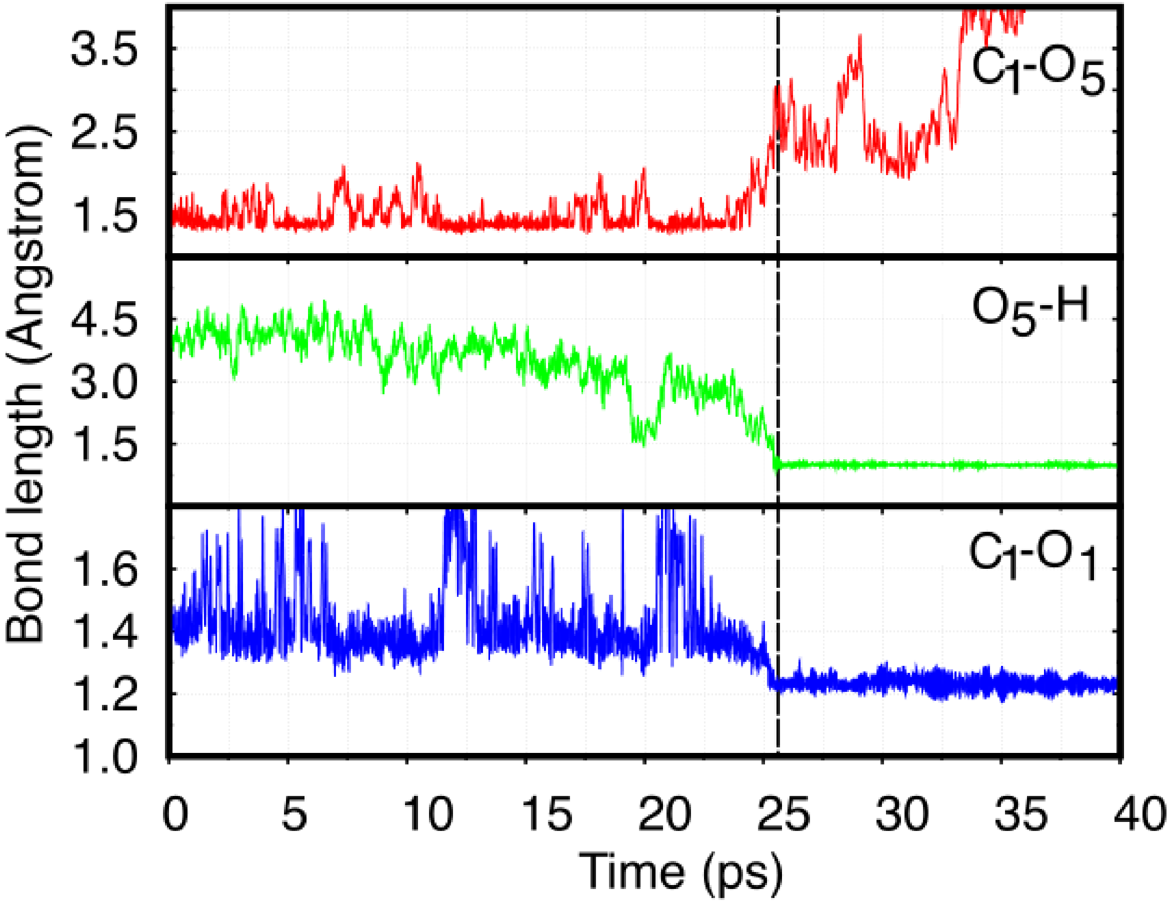

Figure S9: Variation in bond lengths during the metadynamics simulations (in ps) for cyclic α-D-glucose conversion to acyclic form for concerted pathway. CV1 (C_1-O_5) is red; CV2 (O_5-H) is green ; CV3 (C_1-O_1) is blue.

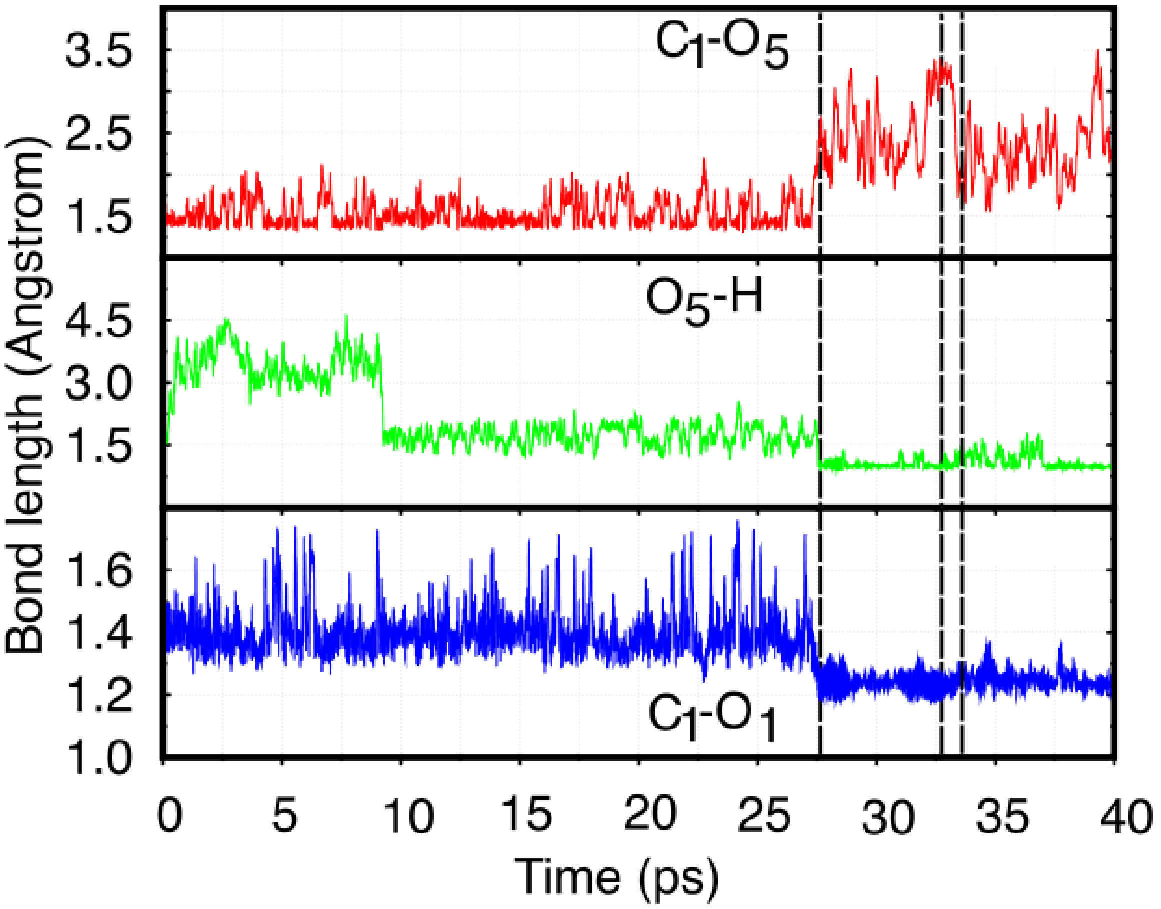

Figure S10: Variation in bond lengths during the metadynamics simulations (in ps) for cyclic β-D-glucose conversion to acyclic form for concerted pathway. C_1–O_5 (CV1) is red; O_5–H (CV2) is green ; C_1–O_1 (CV3) is blue.

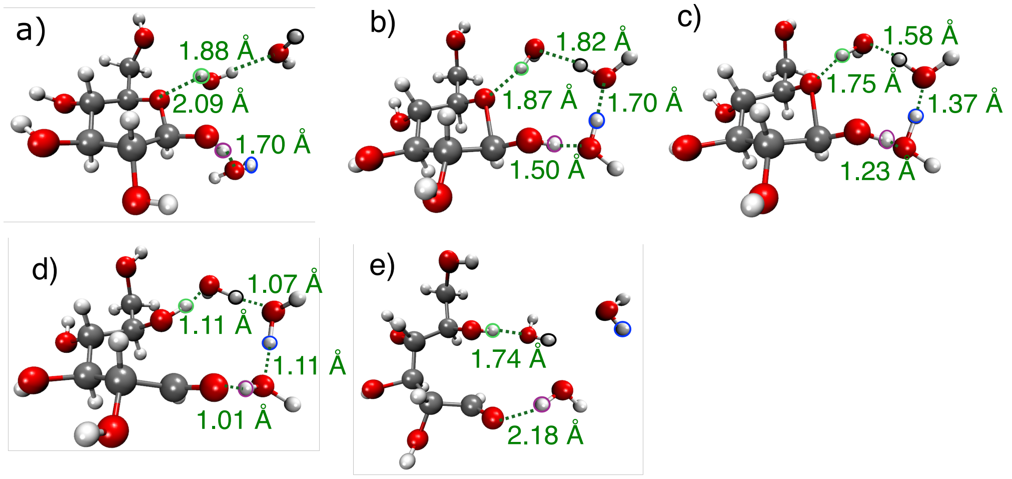

Figure S11: Representative snapshot illustrating the proton relay process involved in the concerted pathway of ring-opening reaction of β-D-glucose at a) 0 b) 27.40 ps c) 27.50 ps d) 27.57 ps e) 29.65 ps. Color circles (purple, blue, black and green) highlight the protons involved.

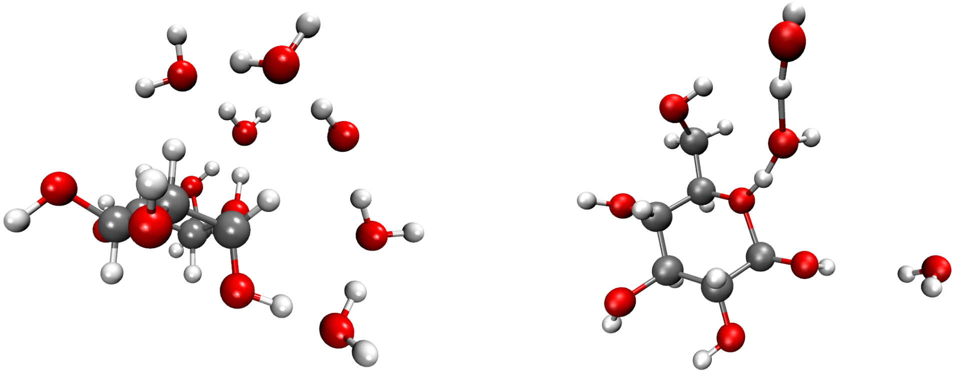

Figure S12: Transition state structures of α- (left) and β-D-glucose (right) for the stepwise pathway highlighting nearby water molecules. In both cases, the solvent does not form a continuous hydrogen-bonded bridge between O_5 and OH_1.

Table S3: Calculated ring strain energy (in kcal/mol) for stepwise and concerted pathways

	Stepwise	Concerted
α-D-glucose	9.7 ± 0.1	11.3 ± 0.1
β-D-glucose	4.1 ± 0.1	10.4 ± 1.5
